# What cell wall components are the best indicators for *Miscanthus* digestibility and conversion to ethanol following variable pretreatments?

**DOI:** 10.1186/s13068-018-1066-3

**Published:** 2018-03-14

**Authors:** J. M. M. Adams, A. L. Winters, E. M. Hodgson, J. A. Gallagher

**Affiliations:** 1 Institute of Biological, Environmental and Rural Sciences, Aberystwyth University, Gogerddan, Aberystwyth, SY23 3EE UK; 2 Present Address: Hodgson Green & Associates, Aberystwyth, UK

**Keywords:** Bioethanol, Biofuel, Biorefining, Cellulose, Dilute acid, Energy crop, *Miscanthus giganteus*, Hemicellulose, Lignocellulosic, Saccharification

## Abstract

**Background:**

Energy crops including *Miscanthus* provide a storable, portable energy source which can be used to complement a wide range of products and energy generation systems. *Miscanthus* is predominantly used in Europe as a combustion material for electricity generation but also has the potential for biochemical conversion due to its high yield and low-nutrient requirements. The ratio of holocellulose (hemicellulose and cellulose combined) to acid detergent lignin (H:L) within the senesced material has previously been shown to indicate the relative suitability of *Miscanthus* accessions for thermochemical conversion. In this study, the ratio was assessed to examine its use as a selection aid for biochemical conversion. 20 highly-characterised *Miscanthus* accessions were saccharified using an enzyme mix to determine optimum sugar release. Nine of these accessions spanning high, medium and low H:L ratios were then autoclaved with dilute acid, alkali or water, and enzymically hydrolysed and fermented to produce ethanol. Samples taken throughout the process allowed assessments of released sugars.

**Results:**

Enzymic degradation of the biomass showed a relationship between H:L ratio and glucose release, with high glucose release for high H:L ratio accessions and vice versa. Xylose release showed no such relationship. This relationship was maintained following pretreatments and enzyme saccharification, where compound analysis showed that following all pretreatments, accessions with high H:L ratios repeatedly had the highest releases of glucose, xylose and arabinose, and produced more ethanol. Release of all measured compounds increased with the pretreatment severity and ethanol yields from each pretreatment correlated with the respective glucose yield, providing assurance that any inhibitory compounds generated were tolerated by the fermentation yeast. Strong correlations were also seen between glucose release, ethanol and cell wall components, with cellulose showing the highest correlations with ethanol yields for some treatments and H:L ratio with others.

**Conclusions:**

The H:L ratio is a good predictor of ethanol yields and sugar release from Miscanthus in this study but individual components lignin and cellulose also correlate well, especially for hot water and mild acid pretreatments. In conclusion, use of the H:L ratio does not provide any advantages over the concentration of individual cell wall components for predicting sugar release and ethanol yields.

## Background

Tackling anthropogenic climate change can only be achieved by replacing fossil-fuel products with a multitude of low-carbon alternatives for generating energy- and carbon-based products. The use of biomass provides a storable, portable energy source which can be processed to complement many existing power and product generation systems. One source of biomass is the ‘energy crops’, high-yielding crops with low moisture content at harvest [1], which grow well on sub-optimal land with low fertiliser demands [2]. Within these requirements, the perennial grasses from Asia in the genus *Miscanthus* [3] have been identified as an energy crop with global potential [2], with some *M. sinensis* hybrids producing up to 41 t Ha^−1^ yr^−1^ [4]. The high-yielding sterile triploid hybrid *Miscanthus x giganteus* has been particularly investigated for its compositional qualities including applications in paper, building materials, geotextiles and greenhouse substrate production [5].

*Miscanthus* harvesting occurs once the plant has senesced (typically following the first autumn frost) and is harvested early the following year. This reduces moisture content, ash and elemental components [1] through the translocation of nutrients from the aboveground plant to the rhizomes during senescence [6]. This produces a feedstock with more desirable biomass processing properties such as a lower reactive alkali metal content and a reduced drying requirement [7], despite a concurrent yield loss compared to peak yield attributed to leaf loss [5], with leaves contributing approximately one-third of the total biomass in *Miscanthus* [8].

Though the predominant use of *Miscanthus* in Europe is currently for energy generation through combustion [7], its high yields mean it should also be considered for biochemical conversion, including the production of liquid biofuels and platform chemicals. To produce these products from *Miscanthus*, the biomass needs to be hydrolysed to release soluble fermentable sugars from the cell wall for conversion by yeast to produce ethanol under anaerobic conditions or other microorganisms to produce a range of chemicals. Spring harvested (senesced) *Miscanthus* will typically contain 70–90% cell wall (w/w) [9, 10], with cellulose, hemicellulose and lignin as the main compounds present. Cellulose and hemicellulose, collectively termed holocellulose, can be hydrolysed to release a number of C5 and C6 sugars with the main forms being xylose and glucose, respectively [3]. Lignin is a complex aromatic polymer which forms a protective barrier within the cell wall around the hemicellulose and cellulose [11], making the lignocellulosic material generally recalcitrant to enzymic conversion [12] unless physiochemical pretreatments are used. Pretreatments include ammonia fibre explosion (AFEX) and other chemical treatments, biological treatments and steam explosion [13]. All treatments alter the lignocellulosic structure [11] by increasing the material porosity, either through the removal of lignin or hemicellulose; or by a reduction in cellulose crystallinity [5]. The ratio of holocellulose to lignin (H:L) has previously been shown to indicate the suitability of *Miscanthus* for thermochemical conversion [14], and work below applied this concept to biochemical conversion, using the ratio as a selection criteria for the *Miscanthus* lines used in this study.

Our research used two common thermochemical pretreatment techniques, dilute acid and alkali pretreatments, both applied with increased temperatures and pressures. Dilute acid is more attractive to industry than concentrated acid due to a reduction in its corrosion, toxicity and inhibitor production [13]. Dilute acid pretreatments primarily hydrolyse the hemicelluloses, though strong acids can also break cellulosic bonds, releasing glucose monomers [15]. Two types of dilute acid pretreatments are typically conducted in studies: a low solids (5–10%), high-temperature (*T* > 160 °C) continuous flow process and a high solids (10–40%), low-temperature (*T* < 160 °C) batch process [11] with work described in this paper categorised as the latter. During acid pretreatments, inhibitors may also be formed which are detrimental to downstream processing [3]. At high temperatures, inhibitors such as furfural can also degrade, forming formic and levulinic acids [13] which further reduce the pH and increase inhibition.

Alkali pretreatments break ester bonds which cross-link lignin and hemicellulose, solubilising lignin molecules and increasing enzyme access to the cellulose [15]. It also partially decomposes the hemicellulose and weakens the hydrogen bonds between cellulose fibrils, reducing the crystallinity and enabling swelling to occur, in turn increasing the surface area of the cellulose [16].

These alkali pretreatments show less sugar hydrolysis than that seen in acid pretreatment processes and have a lower operations cost as the reactor vessel is not subjected to corrosion as with acid [17]. Of the main alkaline pretreatments used (ammonia, sodium, potassium and calcium hydroxide), calcium hydroxide is the least expensive and can be recovered and regenerated using carbon dioxide and established lime kiln technologies [18]. Residues can also be used as soil conditioners with calcium an important macro-nutrient [16]. Calcium hydroxide treatments are effective at delignifying low-lignin biomass though additional reactants are required to remove all lignin in high-lignin biomass [17]. Alkali pretreatments improve the theoretical sugar yield compared with acid pretreatments and so there is a recent increase in focus using this treatment to optimise polysaccharide retention [19]. Thus, both pretreatment processes are of interest to future bioenergy systems.

A number of researchers have conducted studies on *Miscanthus* for sugar release and conversion to ethanol using dilute acid [20], alkali [21] or both [15, 22, 23], sometimes as a two-step procedure [24]. Within these studies different emphases have been put on the *Miscanthus* composition and its role in data interpretation. The majority of yeasts use glucose predominantly or exclusively over C5 sugars such as xylose and arabinose so cellulose availability and degradability are of greater importance in current lignocellulosic biofuel studies, though this is changing. Crystalline cellulose is the main form of cellulose and is created through hydrogen bonds between different layers of glucose polymeric chains and the van der Waals forces between parallel chains [23]. A small proportion occurs as amorphous cellulose and it is more susceptible to hydrolysis in this state [11]. Investigations into cellulose crystallinity and the effect of milling *Miscanthus* on subsequent sugar hydrolysis yields following enzymic degradation showed smaller particle-sized fractions of ball-milled *Miscanthus* had reduced crystalline cellulose in them compared with larger particle-sized fractions. There was also a corresponding increase in glucose release following enzyme addition [25]. Other work determined that lower levels of cellulose crystallinity occurred in *Miscanthus* accessions with high hemicellulose and higher levels in those with high cellulose or lignin proportions. This was an important factor as to why *Miscanthus* with a relatively high hemicellulose content showed high biomass degradation following acid or alkali pretreatments. In contrast, high cellulose or lignin proportions led to lower biomass saccharification occurring, particularly after the acid pretreatment [22]. The reason why high hemicellulose proportions affects the crystallinity of the cellulose was proposed by [15], who in their characterisation studies found that the arabinose substitution degree in the xylan was a key factor relating to degradability following acid and alkali pretreatments. Arabinose is partially associated with amorphous cellulose, so higher levels of arabinose indicated higher amorphous cellulose and, therefore, improved sugar release from the biomass. Cellulose crystallinity can also be reduced through enhancing lignin removal from the biomass through alkali addition which in turn alters the crystallinity of the cellulose [24]. Supporting this, [26] showed that following an alkali pretreatment there was an inverse relationship between lignin content and enzyme hydrolysis yields, making lignin not hemicellulose the main target of the pretreatment.

The most recent review summarising the work surrounding the use of *Miscanthus* for bioethanol production to date is by [27]. They recognise that in addition to the different pretreatments, enzyme saccharifications and ethanol yields are obtained by groups worldwide in this area; the species, cultivation and composition of the *Miscanthus* plants are highly important. A relevant recent article by [28] looked at the main components of plant cells to aid in selection for a range of energy crops, concluding that cellulose content was the main factor in ethanol production.

Following the work of [27] and expanding that of [28], in this study we combine pretreatments and species of variable composition, making it to our knowledge the only manuscript which both uses material from highly-characterised *Miscanthus* lines for conducting dilute acid and alkali pretreatments with complementary hydrothermal pretreatments; and following enzyme hydrolysation produces bioethanol. This study took the known cellulose, hemicellulose and lignin proportions for these samples and calculated the H:L ratio. This was used as a criteria for selecting the analysed *Miscanthus* accessions. Following saccharification alone or pretreatments with acid, alkali or hot water, the glucose and xylose yields were determined from samples taken from pre- and post-enzyme saccharification, with arabinose and ethanol yields also measuring following fermentation. Sampling throughout the experiment has provided insights into the efficacy of the pretreatments themselves and their effect on both subsequent enzyme degradation and ethanol production. This in turn has enabled correlation coefficients to be generated, to examine whether the H:L ratio or other cell wall components are viable indicators for *Miscanthus* digestibility and ethanol production. Insights from this work can be used to aid future *Miscanthus* selection for biochemical conversion.

## Methods

### Miscanthus lines

A collection of 244 different *Miscanthus* genotypes accrued from smaller collections across Europe including those with known origins in China, Japan and South Korea were planted at IBERS, Aberystwyth, in spring 2005. The collection consists of 187× *M. sinensis*, 35× *M. sacchariflorus* and 22× *M.* hybrids, predominantly *M. x giganteus* [2]. In February 2008, material was harvested and milled using a modified silage maize harvester. Samples were dried for 18–24 h at 60 °C then ground through a 1-mm mesh using a hammer mill [3]. A number of analyses have previously been conducted on this material including Neutral Detergent Fibre (NDF), Acid Detergent Fibre (ADF) and Acid Detergent Lignin (ADL), which with ash measurements informed on the proportion of hemicellulose, cellulose and lignin present in each sample [3]. 20 *Miscanthus* genotypes were selected from these accessions using this available composition data and randomly designated 1–20. Selected genotypes included the three different species (*M. giganteus*, *M. sacchariflorus*, *M. sinensis*); and consisted of those with relatively high, intermediate and low holocellulose to lignin ratio contents.

### Enzymic saccharification of non-pretreated *Miscanthus* samples

Enzymic saccharification was conducted as described in [29]. Briefly, 150 mg dry weight milled *Miscanthus* from four biological replicates of the 20 selected genotypes were weighed out in quadruplicate into screwcap glass boiling tubes. Each reaction volume of 10 mL contained sodium citrate buffer (50 mM) and sodium azide (0.02%) with duplicate tubes run with and without enzyme preparations providing approximately 60 FPU g^−1^ cellulase and 64 pNPGU g^−1^ β-glucosidase with additional xylanase at approximately 2500 ABXU g^−1^ xylanase. These were sourced from “mixed cellulase” in Novozymes’ cellulosic ethanol kit vial A (Novozymes), Accellerase 1500 and Accellerase XY (Genencor), respectively. This equated to 2 × 100 μL aliquot additions of 1) Novozyme vial A at a 6 in 7 dilution and 2) Accellerase 1500 and Accellerase XY together, both prepared at a 1 in 10 dilution. Boiling tubes were screwed tight and incubated horizontally at 50 °C ± 1 °C for 168 h at 130 rpm (Gallenkamp shaking incubator). An aliquot of 0.5 mL was removed from each tube following incubation, heated to 100 °C for 10 min then cooled to room temperature before storing at − 20 °C prior to sugar analyses.

### Glucose and xylose determination for non-pretreated samples

Glucose and xylose concentrations of the saccharified *Miscanthus* were determined using d-glucose (GOPOD) and d-xylose assay kits (Megazyme, Bray, Ireland) with the GOPOD assay run at a 1:10 scale and the d-xylose run at microplate scale.

### Severity factor calculations

The *R*_0_ severity factor was determined as described by [30] based on initial definitions by [31]. *R*_0_ = t*exp[*T* − 100/14.75] where *t* = time in minutes and *T* = temperature in °C.

### Acid pretreatment

The acid pretreatment was based on previous acid treatment studies [32]. 3.0 g dry solid-ground *Miscanthus* was added to 60-mL boiling tubes with screwcap lids. Duplicate samples had 20 mL 1% H_2_SO_4_ (w/w) added to a second set of duplicate tubes containing 20 mL deionised water (the mild water pretreatment).

All tubes were vortexed and autoclaved at 111 °C for 30 min with loose lids. Following autoclaving, tubes were cooled to room temperature by incubating in cold water then sampled. A 1 mL aliquot was removed and stored at − 20 °C prior to analysis.

An additional 5 mL was then added to each tube of 0.4 M Ca(OH)_2_ (acid pretreatment samples) or 5.0 mL deionised water (water controls). This generated a pH of approximately 5.0 in each tube following thorough mixing using a clean wire loop.

### Alkali pretreatment

Alkali pretreatment was based on previous work by [18] with a reduced biomass loading to match that used in the acid pretreatment and to enable sample mixing. 3.0 g dry solid-ground *Miscanthus* was added to 60-mL boiling tubes with screwcap lids. Duplicate samples had 20 mL 0.2 M Ca(OH)_2_ added with a second set of duplicate tubes containing 20 mL deionised water (the severe water pretreatment).

All tubes were vortexed and autoclaved at 120 °C for 4 h with loose lids. Following autoclaving, tubes were cooled to room temperature by incubating in cold water then sampled. A 1 mL aliquot was removed and stored at − 20 °C prior to analysis.

An additional volume was added to each tube of either 5 mL 5% (w/w) H_2_SO_4_ (alkali pretreatment samples) or 5.0 mL deionised water (water controls). This generated a pH of approximately 5.0 in each tube following thorough mixing using a clean wire loop.

### Enzyme treatment for pretreated samples

A 0.8 mL enzyme cocktail containing Genencor enzymes Accellerase 1500 (125 μL g^−1^ biomass) and Accellerase XY (50 μL g^−1^ biomass) was added to each tube following the pretreatment and pH adjustments. Tubes were vortexed thoroughly then incubated horizontally with tight lids to maximise enzyme mixing at 50 °C, 150 rpm for 72 h. Following incubation, 1 mL was removed into a microcentrifuge tube, heated to 100 °C for 10 min in a hot block, cooled and frozen for further analysis.

### Fermentation treatment

A 0.5 mL aliquot of a recently prepared 1 in 10 (w/v) Ethanol Red yeast (Fermentis, Marcq-en-Baroeul, France) solution was prepared based on the method in [33] and was used to inoculate each tube. Each tube was briefly vortexed, then incubated at 30 °C with loose lids for 48 h with 1 mL sample taken at termination. All samples were boiled at 100 °C for 10 min, cooled and frozen prior to analysis.

### YSI analysis

A YSI 2700 Select Biochemistry Analyzer (YSI Incorporated, Yellow Springs, OH, USA) was used with glucose and xylose membranes to give selective values for these sugars in the samples taken following the pretreatments and enzyme treatments. The concentrations were calculated using 2700 Xylose PC software LabVIEW 8.5 (YSI Inc.) with subsequent compilations and calculations conducted in Excel (Microsoft Office Professional Plus 2013, Microsoft Redmond, WA, USA).

### High-performance liquid chromatography analysis

To analyse the fermentation products, high-performance liquid chromatography (HPLC) was used based on the method described by [33]. Solutions to be analysed were diluted with 5 mM H_2_SO_4_ containing 5 mM crotonic acid as an internal standard. The mixture was filtered through a 0.45-μm Duropore (PVDF) filtre (Millex-HV, Millipore, USA) and run on a Resex ROA-organic acid H+ column at 35 °C with 5 mM H_2_SO_4_ as the mobile phase at 0.6 mL min^−1^ (Jasco, UK). Concentrations of compounds of interest were determined by refractive index detector and the HPLC software (EZChrom Elite version 3.2, Scientific Software, Agilent Technologies, USA) calibrated with a range of standards. Further calculations were subsequently carried out in Excel (Microsoft).

### Statistical analysis

Data were initially manipulated using Excel (Microsoft), then analysed in IBM SPSS v 23 (IBM Corp) using Multivariate General Linear Model to produce MANOVAs including post hoc Tukey HSD multiple comparison and bivariate correlations generated using Pearson’s product–moment correlation coefficient.

## Results

### Miscanthus feedstock

Twenty selected *Miscanthus* accessions repeated in four adjacent *Miscanthus* trial plots (biological replicates 1–4) were harvested concurrently, weighed, and a sub-sample was dried and milled. A summary table based on dry solids (DS) detailing the *Miscanthus* species, average H:L ratio and composition fractions are presented in Table 1. The composition differences between the highest and lowest lignin and hemicellulose values were 58.9 and 68.7 g kgDS^−1^, respectively, in contrast to cellulose where the difference was 131.6 g kgDS^−1^, showing approximately × 2 greater distribution of the cellulose proportion than within the other main cell wall components. Much of the variation in the holocellulose to lignin ratio can, therefore, be attributed to the cellulose fraction of the holocellulose rather than the hemicellulose or lignin fractions.

**Table 1:** Holocellulose to lignin ratio and composition for selected *Miscanthus* accessions

*Miscanthus* accession	Accession title in second study (if applicable)	Putative species	H:L ratio	Lignin (g kg^−1^ DS)	Cellulose (g kg^−1^ DS)	Hemicellulose (g kg^−1^ DS)	Holocellulose (g kg^−1^ DS)
1	High 1	*sinensis*	10.3	68.7	370.2	335.7	705.9
2		*sinensis*	9.6	76.1	395.0	336.9	731.9
3	High 2	*sacchariflorus*	9.0	77.7	370.1	330.1	700.2
4	High 3	*sinensis*	9.0	91.2	461.8	354.0	815.8
5		*sinensis*	8.7	92.6	458.6	346.0	804.6
6		*sinensis*	8.6	92.8	452.5	341.2	793.7
7		*sinensis*	8.4	92.4	437.6	332.8	770.3
8		*sinensis*	8.0	96.4	446.0	323.9	769.9
9		*sinensis*	7.9	100.0	447.1	339.2	786.4
10		*sacchariflorus*	7.9	97.5	424.2	333.5	757.7
11	Medium 1	*sinensis*	7.7	105.9	474.9	334.3	809.2
12	Medium 2	*sinensis*	7.6	104.8	459.6	338.6	798.1
13	Medium 3	*sacchariflorus*	7.5	102.9	447.5	317.4	764.8
14		*giganteus*	7.4	106.3	462.1	325.5	787.6
15		*giganteus*	6.9	113.5	482.6	291.9	774.5
16		*sinensis*	6.6	114.8	472.5	286.0	758.5
17		*sacchariflorus*	6.5	120.8	470.1	317.6	787.7
18	Low 1	*giganteus*	6.3	124.6	500.1	283.2	783.2
19	Low 2	*sacchariflorus*	6.1	125.8	475.2	295.2	770.4
20	Low 3	*sacchariflorus*	6.0	127.6	449.3	318.4	767.6
Range			6.0–10.3	68.7–127.6	370.1–500.1	283.2–354.0	700.2–815.8
Mean			7.8	101.6	447.8	324.1	771.9

*H:L* holocellulose to lignin ratio, *DS* dry solids

### No pretreatment saccharification assay

**Fig. 1:**
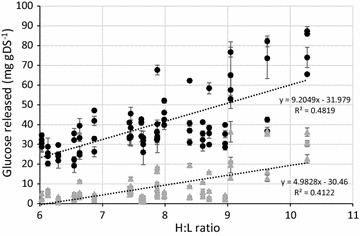
Glucose release from four biological replicates of selected *Miscanthus* accessions following incubation with and without mixed enzyme addition. Black circles = biological replicate incubated with enzymes; grey triangles = biological replicates incubated without enzyme addition. *n* = 2, error bars show standard deviation

Each bag containing a non-pretreated *Miscanthus* accession replicate (biological replicate) was sampled four times, with two technical replicates incubated with additional enzymes and two technical replicates incubated without as controls. Randomly ordered batches of these samples were incubated, following which analysis was conducted to quantify the glucose and xylose released from each sample per gram dry solids. Figure 1 shows the glucose released from each biological replicate with and without enzyme additions against the H:L ratio. Trend lines added for each data set show a general increase in glucose as the H:L ratio increases (*y* = 9.20*x − *31.98, *R*^2^ = 0.48; *y* = 4.98*x *− 30.46, *R*^2^ = 0.41 for values with and without enzyme added, respectively).

Overall, there was a significant difference between biological replicates incubated with enzymes (*P* ≤ .05), with Tukey test analysis showing glucose release from biological replicate 1 was significantly higher (*P* ≤ .01) than replicate 2 though not significantly different from biological replicates 3 and 4. These are shown in Table 2 as mean sugars released and significant differences between replicates are denoted by different lower case letters. There was no significant difference between any of the biological replicates incubated without enzyme additions.

**Table 2:** Glucose and xylose released from selected *Miscanthus* accessions following incubation with and without added enzymes (xylose with enzyme only), separated into replicate plots

Replicate	Glucose released (mg gDS^−1^ ± sd)		Xylose released (mg gDS^−1^ ± sd)
	Enzyme addition	No enzymes	Enzyme addition
1	44.62 ± 19.78 b	10.44 ± 9.76 a	9.15 ± 2.27 a
2	33.82 ± 11.12 a	7.37 ± 6.09 a	9.51 ± 2.18 a
3	41.69 ± 14.72 ab	6.64 ± 9.51 a	9.01 ± 2.41 a
4	39.14 ± 13.57 ab	9.16 ± 9.89 a	9.62 ± 1.32 a

*n* = 40 for each value given. Different lower case letters denote significant differences between treatments calculated using Tukey HSD

*DS* dry solids, *sd* standard deviation

**Fig. 2:**
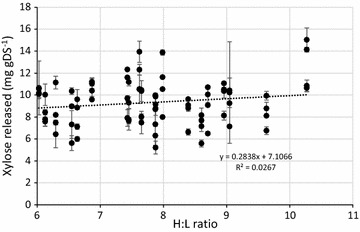
Xylose release from four biological replicates of selected *Miscanthus* accessions following incubation with mixed enzyme addition. Black circles = biological replicate incubated with enzymes. Controls without enzyme addition below detectable limit (< 2 mg gDS^−1^) *n* = 2, error bars show standard deviation

Figure 2 shows the xylose release determined concurrently with the glucose release. Tukey analysis showed no significant difference (*P* > .05) between the four biological replicates incubated with enzyme (Table 2) and there was no alteration of xylose release dependent on the H:L ratio as seen by the generated trend line in Fig. 2 (*y* = 0.28*x* + 7.11, *R*^2^ = 0.03). All untreated samples had a xylose concentration of < 2 mg gDS^−1^, considered below viable quantification in this study.

To carry out a more in-depth analysis, one replicate was selected for a subsequent trial. Replicate 2 has previously been shown [2] to repeatedly give consistent higher biomass yields in the field than the other replicates and so was selected for further study. From replicate 2, nine accessions were studied in greater depth. These covered the range of selected material as before, with relatively high, medium and low holocellulose to lignin ratios (see Table 1) and comprised of accessions 1, 3, 4 (High 1–3); 11–13 (Medium 1–3); 18–20 (Low 1–3).

### Variable pretreatment study

Duplicate technical replicate samples of the replicate 2 *Miscanthus* accessions were randomly grouped prior to analyses as before. Samples underwent acid and alkali pretreatment studies with control water preparations undergoing the same autoclaving conditions. Different methodologies were used for the acid and alkali pretreatments with the logR severity index calculated for each [30, 31]. The acid pretreatment had a severity index of 1.35 and the alkali pretreatment of 2.51. The water comparison samples were, therefore, termed mild and severe water treatments for the acid and alkali conditions, respectively; and were studied alongside the pH-altered samples to determine the relative effect of heat treatment alone on the sugar release and subsequent ethanol production from the *Miscanthus* biomass.

#### Pre- and post-enzymic saccharification

Following the pretreatments, aliquots were removed and analysed for glucose and xylose release. These values are shown in Tables 3 and 4 as “pre-enzyme”, with the MANOVA statistical summary relating to Tukey significant differences within Tables 3, 4, 5 and 6 shown in Table 7. Similar values can be seen for glucose release between each pretreatment and the no-enzyme controls in Fig. 1, showing that the pretreatments alone do not release glucose. The alkali glucose pre-enzyme values are not presented in Table 3 as they were below the detectable limit (BDL), most likely due to detector inhibition [34]. BDL concentrations of xylose were similarly detected in all pre-enzyme samples

**Table 3:** Glucose yields from pretreated samples following enzymic saccharification

Miscanthus accession	Mild water				Severe water				Acid				Alkali		Accession mean
	Pre-enzyme		Post-enzyme		Pre-enzyme		Post-enzyme		Pre-enzyme		Post-enzyme		Post-enzyme		Post-enzyme
High 1	21.1 ± 0.3	c	96.4 ± 3.4	e	18.5 ± 0.7	e	90.3 ± 2.2	e	23.4 ± 1.1	e	136.5 ± 3.4	f	144.6 ± 18.4	ab	75.8
High 2	16.4 ± 4.6	c	78.3 ± 0.8	d	18.6 ± 0.3	ab	87.4 ± 4.6	ab	22.3 ± 0.4	e	127.1 ± 2.8	f	193.3 ± 0.0	c	77.6
High 3	2.7 ± 0.1	a	53.1 ± 1.9	b	3.1 ± 0.3	e	53.6 ± 0.9	e	6.7 ± 0.9	b	112.1 ± 2.2	e	163.5 ± 0.2	abc	56.4
Medium 1	3.7 ± 1.0	ab	55.0 ± 0.9	b	2.3 ± 0.1	a	63.6 ± 3.0	bcd	3.4 ± 0.3	a	85.5 ± 1.4	cd	169.3 ± 4.4	bc	54.7
Medium 2	2.6 ± 0.5	a	61.6 ± 0.5	c	2.4 ± 0.1	a	74.2 ± 0.5	d	3.3 ± 0.5	a	94.1 ± 1.6	d	165.0 ± 5.7	abc	57.6
Medium 3	9.6 ± 1.1	b	58.0 ± 2.6	bc	10.1 ± 1.1	d	66.1 ± 4.9	cd	11.8 ± 0.1	d	81.8 ± 1.5	c	155.8 ± 2.8	ab	56.2
Low 1	3.6 ± 0.2	ab	43.0 ± 1.1	a	3.0 ± 0.2	ab	55.6 ± 0.5	abc	3.9 ± 0.5	a	68.8 ± 3.7	b	144.5 ± 5.1	ab	46.1
Low 2	7.1 ± 0.8	ab	42.4 ± 0.8	a	6.6 ± 0.1	c	50.6 ± 2.2	a	8.4 ± 1.0	bc	56.9 ± 1.6	a	137.2 ± 9.3	a	44.2
Low 3	5.3 ± 0.1	ab	54.4 ± 2.1	b	5.0 ± 0.8	bc	54.0 ± 1.6	ab	9.6 ± 0.4	cd	81.1 ± 4.8	c	138.6 ± 5.4	a	49.7

All values reported in mg g^−1^ dry solids with significant differences as identified by Tukey HSD multiple comparison (different lower case letters denote significant differences between accessions within treatments)

Alkali pre-enzyme = ≤ 2 mg gDS^−1^

*n* = 2 (accession means *n* = 8)

± = standard deviation

**Table 4:** Xylose yields from pretreated samples following enzymic saccharification

Miscanthus accession	Mild water		Severe water		Acid				Alkali		Accession mean
	Post-enzyme		Post-enzyme		Pre-enzyme		Post-enzyme		Post-enzyme		Post-enzyme
High 1	19.6 ± 0.1	e	23.0 ± 2.2	ab	24.1 ± 4.6	ab	95.0 ± 6.9	b	111.0 ± 12.4	a	54.6
High 2	16.4 ± 0.0	cd	23.8 ± 2.0	a	18.4 ± 1.8	ab	74.0 ± 2.5	a	107.8 ± 4.6	a	48.1
High 3	15.1 ± 0.5	bc	16.9 ± 1.0	ab	97.4 ± 13.1	c	155.5 ± 2.8	d	139.3 ± 3.6	b	84.8
Medium 1	15.8 ± 0.9	bcd	21.7 ± 0.2	ab	38.0 ± 5.3	ab	93.6 ± 4.9	b	139.9 ± 7.9	b	61.8
Medium 2	17.5 ± 0.3	de	28.0 ± 0.9	b	40.2 ± 10.1	b	111.5 ± 2.5	c	136.4 ± 1.3	b	66.7
Medium 3	11.3 ± 0.5	a	22.1 ± 3.5	ab	13.5 ± 0.1	ab	61.9 ± 1.4	a	124.2 ± 7.3	ab	46.6
Low 1	10.8 ± 0.2	a	19.9 ± 2.1	ab	12.5 ± 0.6	a	62.3 ± 1.3	a	112.7 ± 1.6	a	43.6
Low 2	13.6 ± 0.1	b	24.9 ± 3.3	ab	28.2 ± 10.4	ab	75.7 ± 3.5	a	130.7 ± 1.7	ab	54.6
Low 3	17.7 ± 1.2	de	18.9 ± 1.8	a	86.0 ± 0.4	c	125.9 ± 4.7	c	126.4 ± 2.4	ab	75.0

All values reported in mg g^−1^ dry solids with significant differences as identified by Tukey HSD multiple comparison (different lower case letters denote significant differences between accessions within treatments)

Mild water, severe water and alkali pre-enzyme values not shown as = ≤ 2 mg gDS^−1^

*n* = 2 (accession means *n* = 8)

± = standard deviation

**Table 5:** Arabinose and ethanol yields from pretreated samples following fermentation

Miscanthus accession	Mild water		Severe water		Acid		Alkali		Accession mean
Arabinose yields ± standard deviation (mg g^−1^ dry solids)									
High 1	21.0 ± 2.7	b	25.8 ± 1.4	b	33.4 ± 6.8	a	55.6 ± 3.5	c	34.0
High 2	14.1 ± 3.4	ab	24.3 ± 6.3	ab	35.6 ± 2.3	a	37.8 ± 2.8	abc	27.9
High 3	15.0 ± 2.7	ab	19.5 ± 1.4	ab	33.5 ± 5.5	a	43.4 ± 9.3	bc	27.9
Medium 1	13.8 ± 0.9	ab	17.5 ± 0.3	ab	27.5 ± 2.3	a	34.2 ± 2.3	ab	23.2
Medium 2	14.7 ± 1.1	ab	17.9 ± 0.2	ab	29.0 ± 2.9	a	34.4 ± 3.1	ab	24.0
Medium 3	13.0 ± 2.1	a	17.0 ± 0.8	ab	27.6 ± 1.9	a	33.6 ± 2.5	ab	22.8
Low 1	12.9 ± 0.4	a	16.1 ± 1.3	a	26.1 ± 2.5	a	29.3 ± 1.4	ab	21.1
Low 2	13.7 ± 0.2	ab	15.8 ± 1.0	a	21.0 ± 1.7	a	19.9 ± 7.5	a	17.6
Low 3	16.2 ± 0.9	ab	20.6 ± 0.8	ab	30.8 ± 6.6	a	46.0 ± 3.7	bc	28.4
Ethanol yields ± standard deviation (μL g^−1^ dry solids)									
High 1	64.9 ± 3.2	d	46.1 ± 9.2	ab	105.0 ± 5.3	g	101.7 ± 0.7	abc	79.4
High 2	36.7 ± 4.9	c	77.8 ± 6.9	a	55.7 ± 4.0	de	151.4 ± 9.6	d	80.4
High 3	27.9 ± 0.4	abc	28.2 ± 0.6	c	74.0 ± 2.4	f	93.6 ± 5.3	ab	56.0
Medium 1	17.5 ± 2.1	a	45.0 ± 3.0	ab	36.6 ± 2.9	bc	119.3 ± 10.6	c	54.6
Medium 2	14.9 ± 1.5	a	50.9 ± 3.8	b	44.3 ± 2.7	cd	121.9 ± 7.7	c	58.0
Medium 3	19.0 ± 9.9	ab	49.5 ± 7.4	b	37.0 ± 3.5	bc	114.3 ± 2.3	bc	54.9
Low 1	14.1 ± 0.5	a	35.1 ± 3.4	ab	28.3 ± 0.7	ab	99.0 ± 1.7	abc	44.1
Low 2	12.4 ± 4.2	a	36.8 ± 5.0	ab	21.7 ± 1.6	a	103.2 ± 1.5	abc	43.5
Low 3	34.9 ± 2.5	bc	30.8 ± 3.2	ab	57.3 ± 1.8	e	85.8 ± 6.6	a	52.2

All values reported (mg and μL g^−1^ dry solids, respectively) significant differences as identified by Tukey HSD multiple comparison analysis (different lower case letters denote significant differences between treatments)

*n* = 2 (accession means *n* = 8)

± = standard deviation

**Table 6:** Comparison of different pretreatments on average subsequent product yields (mg g^−1^ dry solids) *n* = 18

Treatment mean	Post-enzyme glucose		Post-enzyme xylose		Post-enzyme arabinose		Ethanol	
Mild water	60.24	a	15.29	a	14.93	a	26.92	a
Severe water	66.17	a	22.14	a	19.37	a	44.48	b
Acid	93.77	b	95.03	b	29.39	b	51.08	b
Alkali	156.86	c	125.38	c	37.14	c	110.02	c

Significant differences determined using Tukey HSD and shown by different lower case letters

**Table 7:** MANOVA showing main effects of genotype and treatment on sugar release and ethanol yield

Source	Dependent variable	Sum of squares	df	Mean square	F	*P *≤
Genotype	Glucose	17,733.386	8	2216.673	114.390	.000
	Xylose	6431.954	8	803.994	58.934	.000
	Arabinose	1500.168	8	187.521	15.565	.000
	Ethanol	11,367.802	8	1420.975	60.103	.000
Treatment	Glucose	105,577.129	3	35,192.376	1816.078	.000
	Xylose	159,380.491	3	53,126.830	3894.300	.000
	Arabinose	5393.962	3	1797.987	149.238	.000
	Ethanol	70,250.768	3	23,416.923	990.464	.000
Genotype * treatment	Glucose	7025.210	24	292.717	15.105	.000
	Xylose	12,077.095	24	503.212	36.886	.000
	Arabinose	822.883	24	34.287	2.846	.002
	Ethanol	13,493.930	24	562.247	23.781	.000
Error	Glucose	697.616	36	19.378		
	Xylose	491.119	36	13.642		
	Arabinose	433.722	36	12.048		
	Ethanol	851.126	36	23.642		
Total	Glucose	131,033.340	71			
	Xylose	178,380.660	71			
	Arabinose	8150.735	71			
	Ethanol	95,963.626	71			

All samples were then adjusted to pH 5.0 and incubated with a blend of hydrolytic enzymes with activities recommended for lignocellulosic degradation [29] as before. Following incubation, an aliquot of sample was removed to determine the glucose and xylose concentrations as before. Glucose yields, recorded in Table 3 as “post-enzyme”, show a large increase in the glucose release (as high as ×10) for all accessions compared with the pre-enzyme values. In addition, there is an increase of up to 100 mg g^−1^ DS of glucose release in the pH-altered samples compared to the hot water treatment samples. Xylose yields were determined simultaneously with glucose content following enzyme saccharification and are shown in Table 4 as post-enzymic saccharification. As before, these yields have been examined using Tukey HSD multiple comparison analysis and statistically significant differences are shown in Table 3 as different lower case letters; however, the xylose yields showed no clear trends between accessions.

#### Arabinose release and ethanol production

Following enzymic degradation, all *Miscanthus* samples were fermented to produce bioethanol as an example of a bioconversion product. Samples taken after 48 h were analysed for ethanol and arabinose content in addition to glucose and xylose, with yields of ethanol and arabinose shown in Table 5. Ethanol and arabinose proportions increased in samples with pH-altering pretreatments; though there were fewer significantly different yields than with glucose yields.

Glucose presence was not detected in any post-fermentation samples and the xylose concentrations are comparable to post-enzyme levels shown in Table 4; so neither are shown here. Arabinose was also determined following enzymic saccharification and fermentation as it is a significant part of hemicellulose and is also partially associated with amorphous cellulose [15] potentially impacting glucose saccharification yields, but levels showed no decrease during the fermentation period. This is consistent with previous studies by the authors using Ethanol Red yeast which does not ferment xylose or arabinose (data not shown). As before, Tukey HSD was used to determine significant differences in arabinose and ethanol yields between accessions and is shown in Table 5, through different lowercase letters. There is an increase in arabinose release seen in the acid- and alkali-pretreated samples compared to the water pretreatments, but as with the xylose yields, there were no clearly significantly different results for any accession with any of the pretreatments. For the ethanol yields, there was greater clarity, with the acid and alkali pretreatments resulting in higher ethanol yields than the water pretreatments.

#### Pretreatment comparison and correlation analysis

As reported above, there was an increase in sugar release and product yield following acid and alkali pretreatments when compared to the hot water treatments. This was explored further through comparisons of treatment means with Table 6 below clearly showing significantly higher yields for both sugars and ethanol in the acid and alkali pretreatments compared to the hot water treatments. The sequence order for average yields is consistently: ‘mild’ water < ‘severe’ water < acid pretreatment < alkali pretreatment. Significant differences for yields are seen between the water (not significantly different from each other), and acid and alkali pretreatments for all products except ethanol, where severe water was not significantly different to the acid pretreatment, though it was lower.

To explore the relationships between composition, release of sugars and ethanol production further, Pearson’s product-moment correlation was used to generate coefficients between each variable. Previously proposed cellulose:lignin and cellulose:hemicellulose ratios [35] were also included to enable assessment of these ratios too. Correlation analyses considered the influences for each pretreatment, with Table 8 showing correlations ≥ .70 which were also highly significant (*P *≤ .001). A number were to be expected: the H:L ratio was highly correlated to its components, with lignin as the most significant fraction followed by cellulose and hemicellulose; cellulose also correlated to lignin and holocellulose. For other ratios similar component-related correlations were observed: cellulose:hemicellulose correlated with cellulose, hemicellulose, lignin, the H:L ratio and the cellulose:lignin ratio. This latter ratio also correlated with the H:L ratio and lignin.

**Table 8:** Pearson’s product–moment correlation coefficients (≥ .70) between composition components and products generated by enzyme saccharification and fermentation following each pretreatment

	Cell composition and ratios					Pretreatment												
	Cell: Lig	Cell: Hem	Lig	Cell	Hemi	Mild water				Severe water			Acid				Alkali	
						PrE Glu	PoE Glu	PoE Xyl	Eth	PrE Glu	PoE Glu	Arab	PrE Glu	PoE Glu	PrE Xyl	Eth	PoE Glu	PoE Xyl
H:L ratio	.974	− .856	− .986	− .761	.720		.843		.708		.759			.943		.807		
Cell: Lig		− .730	− .939				.727							.864		.736		
Cell: Hem			.882	.889	− .792		− .875	− .732	− .745		− .775	− .783	− .730	− .917		− .781		
Lig				.828		− .710	− .875			− .725	− .827	− .712	− .724	− .944		− .754		
Cell						− .893	− .929		− .823	− .928	− .856	− .840	− .948	− .847		− .708		
Holo				.873		− .926	− .752			− .957	− .728		− .946					.805
Hem														.719				
PrE Glu							.841		.769		.776	.710		.715				
PoE Glu									.851			.727				.861		
PoE Xyl															.932			
Arab									.805					.759				
Eth											.768						.802	

*n* = 18 for all, two-tailed significance ≤ 0.001 for all values below

*Cell* cellulose, *Lig* lignin, *Hem* hemicellulose, *Holo* holocellulose; H:L = holocellulose to lignin ratio, *Cell: Lig* cellulose to lignin ratio, *Cell: Hem* cellulose to hemicellulose ratio, *PrE* pre-enzyme addition, *PoE* post-enzyme addition, *Glu* glucose, *Xyl* xylose, *Arab* arabinose, *Eth* ethanol

Treatments could be separated into distinct groups, with the alkali pretreatment showing just two highly significant correlations: between post-enzyme xylose and holocellulose; and post-enzyme glucose and ethanol. This latter was a key correlation, with all pretreatments showing a significant correlation between the ethanol and post-enzyme glucose values. For the remaining three pretreatments, observed correlations were primarily based on glucose yields, both before and after enzyme addition. Pre-enzyme glucose correlated significantly with compositional components, such as lignin, cellulose and holocellulose (and cellulose:hemicellulose for the acid pretreatment only), reflecting the main inhibitors to cell wall degradation and the main glucose-generating substrates. Post-enzyme glucose yields also correlated with these components (except the acid pretreatment which did not correlate with holocellulose), but also correlated with the H:L, cellulose:hemicellulose and cellulose:lignin (not severe water) ratios, pre-enzyme addition glucose and ethanol yields. The last set of correlations were primarily based on the ethanol yield which correlated with H:L, cellulose:hemicellulose ratios and cellulose for the mild water and acid pretreatments, with the acid pretreatment also correlating to cellulose:lignin and lignin. In addition, arabinose correlated with the cell wall components and cellulose:hemicellulose ratio (severe water pretreatment only), pre- and post-enzyme glucose yields for the severe water pretreatment and post-enzyme glucose (acid pretreatment).

## Discussion

The aim of this research was to assess whether using the H:L ratio provided an improved indicator for biological conversion suitability, in particular for biomass degradability and ethanol generation compared with lignin or other cell wall components. This builds on the concept proposed by [14] that the H:L ratio correlates with the relative suitability of *Miscanthus* for pyrolysis and combustion routes in thermochemical conversion. Similar correlations were identified by [35] for biological conversion who found that ratios of cellulose:lignin and cellulose:xylan could be major determinants of *Miscanthus* biomass degradability. The Pearson’s product of moment correlation coefficient for these ratios with sugar release and ethanol product was determined to assess their potential as indicators too.

Samples of 20 highly-characterised *Miscanthus* accessions with high, medium and low H:L ratios were analysed for sugar release with and without added enzymes. Following enzyme addition, there was a general trend seen with an increase of glucose release occurring as the H:L ratio increased. No such trend was observed for xylose, suggesting that the cellulose proportion of the H:L ratio was the key component in the holocellulose fraction regarding lignocellulose degradation.

To investigate this further, a second study was initiated with nine selected accessions undergoing pretreatments prior to enzyme saccharification and conversion to ethanol. Here, the trend relating glucose yield to H:L and a lack of correlation with the xylose was repeated. Accessions High 1 and High 2 repeatedly showed significantly higher glucose release yields than the other accessions following pretreatments with acid or hot water produced by ‘mild’ or ‘severe’ autoclaving; before and after enzyme addition. These accessions also showed distinct patterns of arabinose release and ethanol production; High 1 gave higher arabinose release than the other accessions, following all pretreatments, but not significantly so. In contrast, for ethanol production there was a clearer split, with High 1 showing significantly higher yields than the other accessions following the mild water and acid pretreatments. High 2 was significantly higher than High 1 for the severe water and alkali-pretreated samples. For both accessions, the yield was considerably higher, with approximately 25 and 50 μL more ethanol produced per g DS than the second highest yielder for both water treatments and pH-altered pretreatments, respectively.

Yields of sugar release from the biomass following the hot water pretreatments both before and after enzyme addition were comparable to those seen in Fig. 1 from the first study, where the samples were not pretreated. This suggests that the biomass itself did not alter significantly during the autoclaving process and that structural alteration only occurred at higher temperatures. This reflects that which has been observed in previous studies, for example in the work by [36] where they showed consistent values for glucose yield following enzyme hydrolysis and cellulose fibril crystallinity following hydrothermal reactions between 50 and 155 °C. Above this temperature (assessed to 200 °C), fibril crystallinity decreased; glucose yields following enzyme additions increased. In contrast, samples undergoing acid or alkali pretreatments at high temperature did show significant increases in sugar release compared with the water controls and those in the initial study, showing that structural changes occurred.

This is succinctly shown in Table 6 which shows significant differences between pretreatments when the means of all nine accessions are combined. The sequence ‘mild water’ < ‘severe water’ < acid < alkali pretreatments on glucose, xylose, arabinose and ethanol yields shows that though there was less variation in sugar release and product generation between accessions with the alkali pretreatment, yields were the highest for sugar release and ethanol yields, making this potentially a process to employ on mixed-origin *Miscanthus* requiring degradation to downstream processing including second-generation biofuels. Conversely, greater variation was seen between accessions with the water and acid pretreatments, which due to their low costs [37] are more likely to be utilised for biochemical conversion routes, and could be a preferred conversion route for characterised *Miscanthus* with known composition.

A comparative examination of all data values to assess the indicative value of the H:L ratio versus lignin or other cell wall component proportion or ratio on sugar and ethanol yields was conducted using Pearson’s product of moment correlations (Table 8). Within the table, certain correlations are as predicted. All main cell components showed highly significant correlations with each other, the ratios generated and with the compounds produced, especially those undergoing the acid pretreatment and hot water conditions. The other main correlations were between the glucose yield (particularly post-enzyme saccharification) and ethanol, again unsurprisingly as glucose constituted the almost exclusive substrate for the yeast in this study. Arabinose did correlate, but considering its potential role in indicating cellulose crystallinity its correlations showed no clear pattern and are considered to be indirectly related compared to those above as this sugar is not utilised by the yeast. The correlation with glucose also indicated that the levels of any inhibitors such as furfural and hydroxymethyl furfural which may have been generated by the pretreatments were tolerated by the yeast. This is a key concern in the downstream processing of biomass to biofuels as high levels of inhibitors can have a significant effect on the yeast, reducing subsequent yields of bioethanol produced [17]. A second point of concern regarding the pretreatments is that a balance must be struck between the pretreatment severity and product yield. Pretreatments which aggressively remove or redistribute lignin also result in sugar loss through degradation [38]; so this study intentionally used optimal ‘mild’ acid and alkali pretreatments to minimise the degradation of sugars whilst maximising the differences seen between the *Miscanthus* genotypes.

## Conclusion

The original objective was to attempt to determine which component or combination (e.g. H:L ratio) is the most accurate for determining sugar release and ethanol yields from *Miscanthus*. H:L ratios show a higher correlation with ethanol than lignin for mild water and acid-pretreated samples (severe water and alkali-pretreated samples showed < 0.7 correlation for both H:L and lignin); but lignin shows a higher correlation to post-enzyme glucose than H:L for both hot waters and acid pretreatments (alkali pretreatment had < 0.7 correlation) and post-enzyme glucose shows the highest correlation with ethanol following all pretreatments. If the post-enzyme glucose was the focus, then cellulose is the highest correlating cell wall component with post-enzyme glucose and the highest correlating cell wall component with ethanol produced under ‘mild water’ conditions; we propose that this is used as the predictive component for hot water pretreatments. The H:L ratio correlates most highly to ethanol for the acid pretreatments, but cellulose:hemicellulose, cellulose:lignin, lignin and cellulose also correlate highly. We, therefore, propose that no additional advantage is given through the determination of multiple cell wall components to produce ratios over single cell wall components. We also conclude that in addition to lignin, cellulose alone can be used as a predictor for *Miscanthus* conversion by glucose-utilising yeast to ethanol following all pretreatments but especially those of hot water and mild acid.

## Abbreviations

BDL: below detectable limit
H:L: holocellulose to lignin ratio
DS: dry solids
